# A methylbenzimidazole derivative regulates mammalian circadian rhythms by targeting Cryptochrome proteins

**DOI:** 10.12688/f1000research.124658.1

**Published:** 2022-09-07

**Authors:** Moeri Yagi, Simon Miller, Yoshiko Nagai, Shinsuke Inuki, Ayato Sato, Tsuyoshi Hirota

**Affiliations:** 1 Institute of Transformative Bio-Molecules, Nagoya University, Nagoya, 464-8601, Japan; 2 Division of Biological Sciences, Graduate School of Science, Nagoya University, Nagoya, 464-8601, Japan; 3 Graduate School of Pharmaceutical Sciences, Kyoto University, Kyoto, 606-8501, Japan

**Keywords:** Circadian clock, Cryptochrome, Small-molecule compound

## Abstract

**Background**: Impairment of the circadian clock has been associated with numerous diseases, including sleep disorders and metabolic disease. Although small-molecules that modulate clock function may form the basis of drug discovery of clock-related diseases, only a few compounds that selectively target core clock proteins have been identified. Three scaffolds were previously discovered as small-molecule activators of the clock protein Cryptochrome (CRY), and they have been providing powerful tools to understand and control the circadian clock system. Identifying new scaffolds will expand the possibilities of drug discovery.

**Methods**: A methylbenzimidazole derivative TH401 identified from cell-based circadian screens was characterized. Effects of TH401 on circadian rhythms were evaluated in cellular assays. Functional assays and X-ray crystallography were used to elucidate the effects of the compound on CRY1 and CRY2 isoforms.

**Results**: TH401 lengthened the period of circadian rhythms and stabilized both CRY1 and CRY2. The compound repressed *Per2* reporter activity, which was reduced by *Cry1* or *Cry2* knockout and abolished by *Cry1/Cry2* double knockout, indicating the dependence on CRY isoforms. Thermal shift assays showed slightly higher interaction of TH401 with CRY2 over CRY1. The crystal structure of CRY1 in complex with TH401 revealed a conformational change of the gatekeeper W399, which is involved in isoform-selectivity determination.

**Conclusions**: The present study identified a new small-molecule TH401 that targets both CRY isoforms. This compound has expanded the chemical diversity of CRY activators, and will ultimately aid in the development of therapeutics against circadian clock-related disorders.

## Introduction

Living organisms have a molecular clock, called the circadian clock, which drives the ~24-hour circadian rhythm. The circadian clock regulates daily rhythms of various physiological processes, such as sleep-wake behavior, body temperature, and metabolism. ^1^ This clock is composed of a transcriptional regulatory network of clock genes, *Period* ( *Per1* and *Per2*), *Cryptochrome* ( *Cry1* and *Cry2*), *Clock* and *Bmal1.* In the core feedback loop of the mammalian circadian clock, transcription factors CLOCK and BMAL1 activate transcription of *Per* and *Cry* genes by forming a heterodimer. The translated PER and CRY proteins then repress the transcriptional activity of CLOCK-BMAL1 to close the loop, followed by the degradation of PER and CRY through the ubiquitin-proteasome pathway reactivating CLOCK-BMAL1. ^2^

Impairment of clock functions due to genetic mutations of clock genes or environmental factors, including shift work or chronic jet lag, has been shown to cause sleep disorders and increase the risk of numerous diseases, such as obesity and cancer. ^3^ Thus, elucidating the circadian clock system is important for understanding how circadian clock dysfunction results in circadian-related diseases. Small-molecule compounds that control clock function provide a powerful and useful tool in the drug discovery of such diseases. ^4^^–^^6^ Cell-based chemical screening has identified several synthetic small-molecule compounds that selectively target the core clock protein CRY. A carbazole-containing compound KL001 targets both CRY1 and CRY2 to inhibit their ubiquitin-dependent degradation, thus lengthening the circadian period. ^7^ Several KL001 derivatives have been developed, including KL044 which is 10 times more potent than KL001, ^8^ and a period-shortening compound GO044. ^9^ Several other KL001 derivatives have shown potential application in the treatment of diabetes and glioblastoma. Compound **41** and compound **50** improved glucose clearance in diet-induced obese mice and *db/db* mice, respectively, indicating their antidiabetic efficacy. ^10^^,^^11^ Treatment with KL001 and its derivative SHP656 inhibited proliferation and survival of patient-derived glioblastoma stem cells (GSCs), which cause a highly malignant primary brain tumor, and SHP656 prolonged the survival of mice implanted with GSCs. ^12^ Furthermore, a new series of CRY activators that target either CRY1 or CRY2 in an isoform-selective manner were recently identified: phenylpyrazole-containing compounds KL101, TH301, and TH129, ^13^^,^^14^ and a thienopyrimidine derivative KL201. ^15^ In addition to these three scaffolds, the identification of novel scaffolds will expand the chemical diversity of CRY activators, as well as the possibility of drug discovery for the treatment of circadian clock-related diseases.

In this study, we revealed the effects of a new circadian clock modulator TH401, which contains a methylbenzimidazole moiety, on CRY isoforms by taking a target-based approach. TH401 showed stabilization and activation of CRY1 and CRY2. The repression of *Per2* reporter by TH401 was dependent on both CRY isoforms, indicating CRY-specific activity of the compound. TH401 directly interacted with CRY1 and CRY2, albeit with a slight preference to CRY2, and the X-ray crystal structure of a CRY1-TH401 complex revealed the binding mode of TH401.

## Methods

### TH401 and derivatives

TH401 powder was purchased from Vitas-M Laboratory (STK095604). TH403-TH411 were obtained from a 10 mM original stock of a compound library containing 20,000 small molecules used for primary screening of circadian clock modulators.

### Cell-based circadian assays

U2OS cells expressing a *Bmal1-dLuc* and *Per2-dLuc* reporter ^16^^,^^17^ were plated onto a white, solid-bottom 384-well plate at 30 μl (3,000 cells) per well as previously described. ^18^ After 2 days, 40 μl of explant medium [DMEM (12800-017, Gibco) supplemented with 2% B27 (17504-001, Gibco), 10 mM HEPES, 0.38 mg/ml sodium bicarbonate, 0.29 mg/ml L-glutamine, 100 units/ml penicillin, 100 μg/ml streptomycin, and 1 mM luciferin; pH 7.2] was dispensed into each well, and 500 nl of compounds (final 0.7% dimethyl sulfoxide (DMSO)) were applied. The luminescence was recorded every 100 min for 5 days in a microplate reader (Infinite M200Pro, Tecan).

### Cell viability assay

*Bmal1-dLuc* and *Per2-dLuc* U2OS cells were plated by following the cell-based circadian assay protocol as described above and cultured for 5 days. Then, CellTiter-Glo Reagent (G9242, Promega) was applied to each well, and luminescence corresponding to cellular ATP levels was recorded in a multi-mode reader (Cytation3, BioTek).

### *In vitro* kinase assay

The effect of compounds on casein kinase Iδ (CKIδ) activity *in vitro* was analyzed as previously described. ^19^ The reaction mixture containing 2 ng/μl CKIδ (14-520, Eurofins), 50 μM peptide substrate RKKKAEpSVASLTSQCSYSS corresponding to human PER2 Lys659-Ser674 (custom made), CKI buffer (40 mM Tris, 10 mM MgCl _2_, 0.5 mM DTT, 0.1 mg/ml BSA, pH7.5), compound (final 5% DMSO), and 5 μM ATP was incubation at 30°C for 3 h. Kinase-Glo Luminescent Kinase Assay reagent (V6713, Promega) was used to determine the amount of remaining ATP.

### Degradation assay

Stable HEK293 cells expressing a C-terminally luciferase-fused CRY1 (CRY1-LUC), CRY2-LUC or LUC reporter were plated onto a white, solid-bottom 96-well plate (30,000 cells per well) and treated with TH401 for 24 h as previously described. ^7^ After 24 h treatment with compounds, luciferin (final 0.1 mM) and HEPES-NaOH (pH 7.2; final 10 mM) were added to the medium. After 1 h, it was further supplemented with cycloheximide (final 20 μg/ml), and luminescence was recorded every 10 min for 18 h in a microplate reader (Infinite M200Pro, Tecan).

### *Per2::Luc* repression assay

Wild type, *Cry1/Cry2* double knockout, *Cry1* knockout, and *Cry2* knockout fibroblasts expressing a *Per2::Luc* knock-in reporter ^20^ were plated on a white, solid-bottom 384-well plate. They were cultured for 2 days to reach confluency, and 500 nl of compounds (final 0.7% DMSO) were applied. After 2 days of treatment with the compounds, the medium was replaced with BrightGlo (E2620, Promega), and luminescence was recorded in a multi-mode reader (Cytation3, BioTek).

### *Cry* rescue assay

Functional rescue of *Cry1/Cry2* double knockout mouse embryonic fibroblasts with CRY expression vectors ^21^ was performed as previously described ^13^ with modifications: 15,000 cells were plated onto a white, solid-bottom 96-well plate, and after 24 h, transfected with 0.1 or 0.2 ng of CRY1 and CRY2 expression vectors and 100 ng of a *Bmal1-Eluc* reporter vector by Fugene 6 (E2691, Promega). After treatment with forskolin (final 10 μM) for 2 h, the medium was replaced with explant medium containing 0.2 mM luciferin, and 500 nl of compounds (final 0.4% DMSO) were applied. Luminescence was recorded every 36 min in a microplate reader (Infinite M200Pro, Tecan) for 5 days.

### Cellular thermal shift assay

HEK293T cells were co-transfected with Flag-tagged CRY1 and HA-tagged CRY2 expression vectors as previously described. ^13^ After 2 days, the cell pellet was suspended in serum-free DMEM with cOmplete EDTA-free Protease Inhibitor Cocktail (04693132001, Roche), treated with 0, 8, and 24 μM of compounds (final 0.7% DMSO) on a 96-well PCR plate, and incubated at 37°C for 1 h, followed by heat treatment for 3 min. The optimized temperatures for heat treatment of CRY1 and CRY2 were 55°C and 49°C, respectively. The cells were lysed by 2 cycles of freeze-thawing and centrifuged at 18,000 x g for 20 min at 4°C. The supernatants were analyzed by Western blotting with mouse monoclonal anti-Flag-HRP (A8592, Sigma; RRID: AB_439702) and rat monoclonal anti-HA-HRP (12013819001, Roche; RRID: AB_390917) antibodies.

### Recombinant CRY expression and purification

His _6_-MBP-CRY1(PHR) and His _6_-MBP-CRY2(PHR) were expressed in Sf9 ( *Spodoptera frugiperda*) insect cells (Invitrogen) via baculovirus infection as previously described. ^13^ Cell pellets were resuspended in lysis buffer (1x PBS, 50 mM NaNO _3_, 1% (v/v) glycerol, 0.1% Triton X-100, and Complete Protease Inhibitor Cocktail (Roche); pH 7.4) and purified according to our previously determined method. ^14^ Briefly, cells were sonicated on ice, centrifuged at 19,000 × g for 90 min at 4°C, and the supernatant, containing target CRY proteins, was purified via a high-performance liquid chromatography (HPLC) system using a HisTrap 5 ml column (GE Healthcare). After tobacco etch virus (TEV) protease cleavage of the His _6_-MBP tag, further purification was performed via a HiTrap Heparin HP column (GE Healthcare), amylose resin (E8021, New England Biolabs), and a gel filtration chromatography Superdex 75 16/60 column (GE Healthcare). Purified proteins were buffer-exchanged (see Protein crystallization and structure determination section) and concentrated using an Amicon Ultra (Merck) concentrator.

### Thermal shift assay

CRY1(PHR) or CRY2(PHR) were diluted to 2 μM with differential scanning fluorimetry (DSF) buffer (20 mM HEPES-NaOH, 150 mM NaCl, 2 mM DTT; pH 7.5) and dispensed into a 384-well white PCR plate (Bio-Rad) at 17 μl per well. After the application of 1 μl of compounds (final 5% DMSO), the mixtures were incubated at room temperature with gentle shaking for 60 min. 2 μl of SYPRO Orange (S6650, Invitrogen) diluted with DSF buffer (final 5x SYPRO Orange) was added, and thermal denaturation was performed using a real-time PCR detection system (CFX384 Touch, Bio-Rad).

### Protein crystallization and structure determination

CRY1(PHR) was buffer-exchanged into 100 mM Bis-Tris propane (B6755, Sigma), 100 mM NaCl, and 2 mM tris(2-carboxyethyl) phosphine (209-19861, Wako Pure Chemical Industries); pH 7.5, concentrated to 6 mg/ml, and crystallized via hanging-drop vapor diffusion at 20°C. CRY1(PHR) (1 μl) was mixed with 1 μl of precipitant solution containing 250 mM NH _4_Cl, 21% (w/v) PEG 3350, 3% (v/v) ethylene glycol. Apo crystals grew over several days and were soaked overnight with 0.5 mM TH401 dissolved in mother liquor. The crystals were cryoprotected in mother liquor plus 30% (v/v) PEG 400, and flash-cooled in liquid nitrogen. In contrast, we were unable to obtain protein crystals of a CRY2-TH401 complex.

X-ray diffraction data for CRY1-TH401 was collected at the SPring-8 synchrotron radiation facility (beamline BL41XU) at a wavelength of 1.0 Å and a temperature of 100 K. The dataset was processed with DIALS/xia2 ^22^ and SCALA ^23^ in the CCP4 suite. ^24^ The CRY1-TH401 structure was determined in space group P2 _1_2 _1_2 _1_ (1 molecule per asymmetric unit) by Phaser ^25^ using CRY1-apo (PDB ID: 6KX4) as a molecular replacement (MR) template. Density modification was performed with PARROT. ^26^ Model building was performed iteratively using Coot ^27^ and refinement in REFMAC5. ^28^ Final refinement was performed with PHENIX refine. ^29^

### Quantification

A curve fitting program MultiCycle (Actimetrics) was utilized to determine the circadian period, and the luminescence intensity was calculated by averaging the intensity during the entire experiment. Due to transient changes in luminescence upon medium exchange, data from the first day was excluded from analysis. In degradation assays, half-life was obtained by one phase exponential decay fitting with Prism software (version 7.04, GraphPad Software; any open-access software can be used as an alternative, including the freely available R). In cellular thermal shift assays, band intensity was analyzed by ImageQuant TL software (version 8.1, GE Healthcare). In thermal shift assays, the highest peak of the dF/d *T* curve (the first derivative of the fluorescence intensity against temperature) was defined as the melting temperature.

## Results and discussion

### TH401 lengthens circadian period

**Figure 1.**
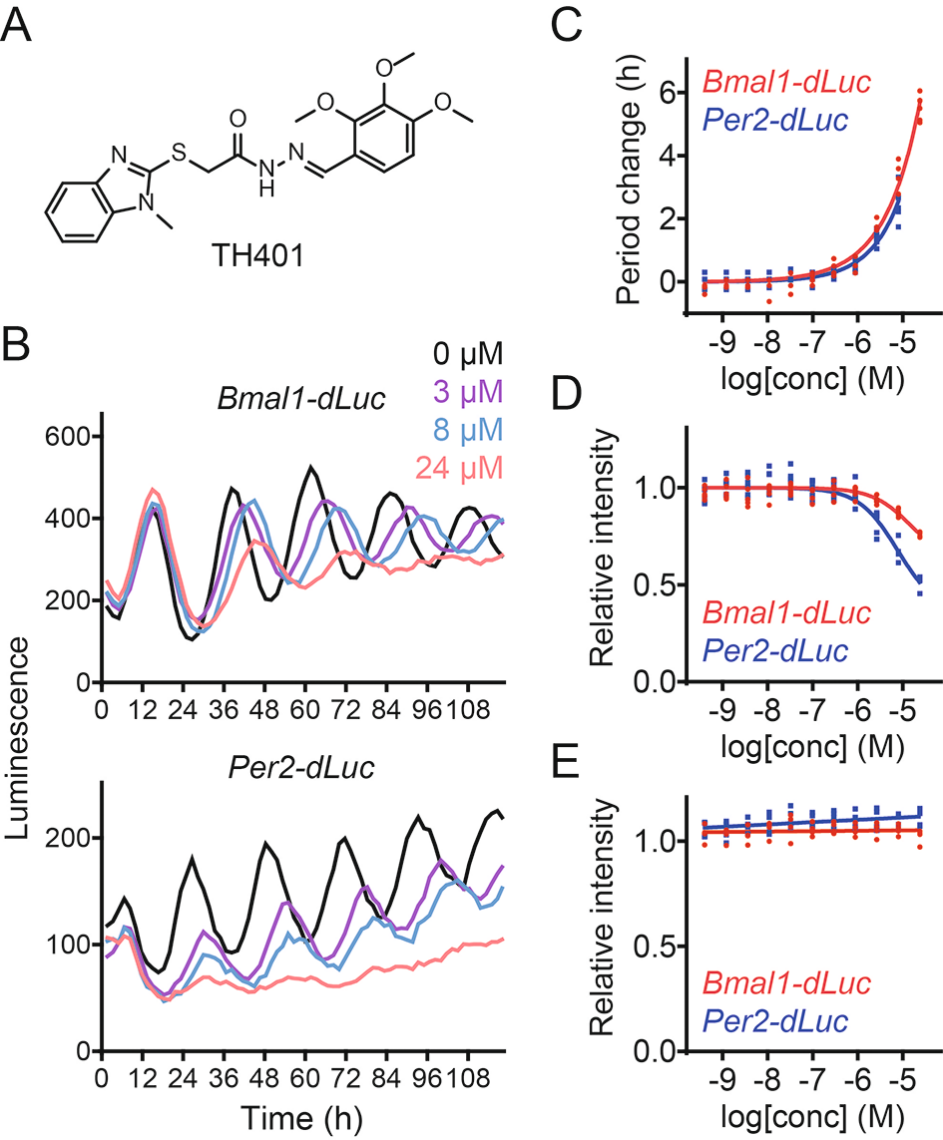
TH401 lengthens circadian period. (A) The chemical structure of TH401. (B-D) Effects on circadian rhythms in *Bmal1-dLuc* and *Per2-dLuc* U2OS cells. Luminescence rhythms in the presence of various concentrations of TH401 (B, mean of *n* = 2) and changes in period (C) and luminescence intensity (D) compared to a dimethyl sulfoxide (DMSO) control are shown ( *n* = 6 biologically independent samples). When arrhythmic, the period is not plotted. (E) Effect on cell viability in *Bmal1-dLuc* and *Per2-dLuc* U2OS cells. Cellular ATP levels after treatment with various concentrations of TH401 are plotted by setting a DMSO control to 1 ( *n* = 4 biologically independent samples).

We discovered new small-molecule modulators of the circadian clock from cell-based screens of a library of ~20,000 uncharacterized compounds. ^13^^,^^14^^,^^18^ In this study, we characterized a methylbenzimidazole derivative TH401 ( Figure 1A). Treatment of human U2OS cells expressing either a *Bmal1* promoter-luciferase ( *Bmal1-dLuc*) reporter or a *Per2-dLuc* reporter with TH401 caused lengthening of the circadian period in a dose-dependent manner ( Figure 1B and C). ^30^ Furthermore, increasing the concentrations of TH401 suppressed the intensity of the *Per2-dLuc* reporter more than that of *Bmal1-dLuc* ( Figure 1B and D), ^30^ without affecting cellular viability ( Figure 1E). ^30^ These results indicate that TH401 is a new clock-modulating compound.

### TH401 targets both CRY isoforms

**Figure 2.**
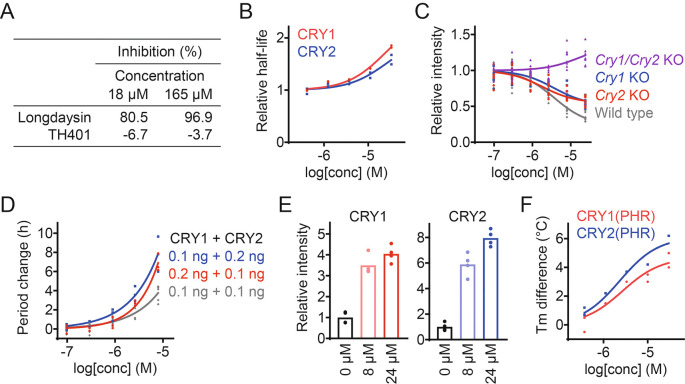
TH401 stabilizes and interacts with CRY1 and CRY2. (A) Effect of TH401 on casein kinase Iδ (CKIδ) activity *in vitro.* Kinase activity was analyzed in the presence of various concentrations of compounds ( *n* = 1). Longdaysin is an inhibitor of CKIδ. (B) Effect of TH401 on Cryptochrome (CRY) degradation in HEK293 cells. The half-lives of CRY-luciferase fusion proteins (CRY1-LUC and CRY2-LUC) relative to LUC are plotted by setting a DMSO control to 1 ( *n* = 2 biologically independent samples). (C) Effect on *Per2::Luc* knock-in reporter activity in wild type, *Cry1/Cry2* double knockout, *Cry1* knockout, and *Cry2* knockout fibroblasts. Changes in luminescence intensity compared to a DMSO control are shown ( *n* = 4–8 biologically independent samples). (D) Effect on cellular circadian period of *Bmal1-Eluc* reporter rhythms in *Cry1/Cry2* double knockout fibroblasts rescued with CRY. Changes in period compared to a DMSO control are shown ( *n* = 3–6 biologically independent samples). (E) Interaction with CRY proteins in HEK293T cells. The band intensities of Flag-tagged CRY1 and HA-tagged CRY2 proteins protected from thermal denaturation in cells are plotted by setting a DMSO control to 1 (mean of *n* = 4 biologically independent samples). Compound interaction induced thermal stabilization. (F) Interaction with CRY1(PHR) and CRY2(PHR) *in vitro.* Changes in denaturing temperatures of recombinant CRY(PHR) proteins in the presence of various concentrations of TH401, compared to a DMSO control are shown ( *n* = 2 biologically independent samples).

We took a target-based approach to reveal how TH401 modulates circadian rhythms. Longdaysin is known to induce period lengthening by targeting the protein kinase CKIδ, ^19^ but TH401 did not affect CKIδ activity in an *in vitro* kinase assay ( Figure 2A), ^30^ suggesting an alternative mechanism of action other than CKI. We next analyzed the effect of TH401 on CRY stability in a cell-based degradation assay. HEK293 cells expressing a CRY1-luciferase (CRY1-LUC) or CRY2-LUC fusion protein reporter were treated with the compound at various concentrations, and the half-life of luminescence signals were measured. TH401 stabilized both CRY1 and CRY2 ( Figure 2B), ^30^ suggesting that the compound targets CRY proteins.

The effect of TH401 on endogenous CRY1 and CRY2 activity was analyzed by using *Cry* knock-out fibroblasts from mice carrying a *Per2::Luc* knock-in reporter. ^31^ CRY is a repressor of CLOCK-BMAL1, and CRY stabilization reduces the expression of CLOCK-BMAL1-target genes such as *Per2*. ^2^ TH401 repressed the intensity of the *Per2::Luc* reporter in a dose-dependent manner in wild type cells with both CRY1 and CRY2 present ( Figure 2C). ^30^ *Per2* repression was not observed in *Cry1/Cry2* double knockout fibroblasts, indicating that the effect of TH401 was CRY-dependent. In *Cry1* and *Cry2* single knockout cells, *Per2* repression by TH401 was reduced compared to wild type, which supports that TH401 targets both CRY1 and CRY2. We further evaluated its effect on the circadian period of a *Bmal1-Eluc* reporter in *Cry1/Cry2* double knockout mouse fibroblasts rescued with CRY1 and CRY2. Period-lengthening by TH401 was enhanced when the dose of CRY1 or CRY2 was increased ( Figure 2D). ^30^

To assess the interaction of TH401 with CRY proteins, a cellular thermal shift assay was conducted using HEK293T cells expressing CRY1-Flag and CRY2-HA. Exposing proteins to a high temperature causes them to lose their tertiary structure. However, the binding of a ligand increases resistance to unfolding, leading to thermal stabilization of the bound protein. ^32^ TH401 stabilized CRY1 and CRY2 against thermal denaturation in a dose-dependent manner ( Figure 2E), ^30^ suggesting that TH401 interacts with both CRY isoforms. The direct interaction of TH401 with recombinant CRY1 photolyase homology region (PHR) and CRY2(PHR) was further evaluated by performing an *in vitro* thermal shift assay. We found that TH401 interacted with both recombinant CRY(PHR) proteins with a slightly higher preference against CRY2 over CRY1 ( Figure 2F). ^30^ Together, these data indicate that TH401 induces circadian period lengthening by targeting and interacting with both CRY1 and CRY2 proteins.

### Structural binding mechanisms of TH401 in CRY1

**Figure 3.**
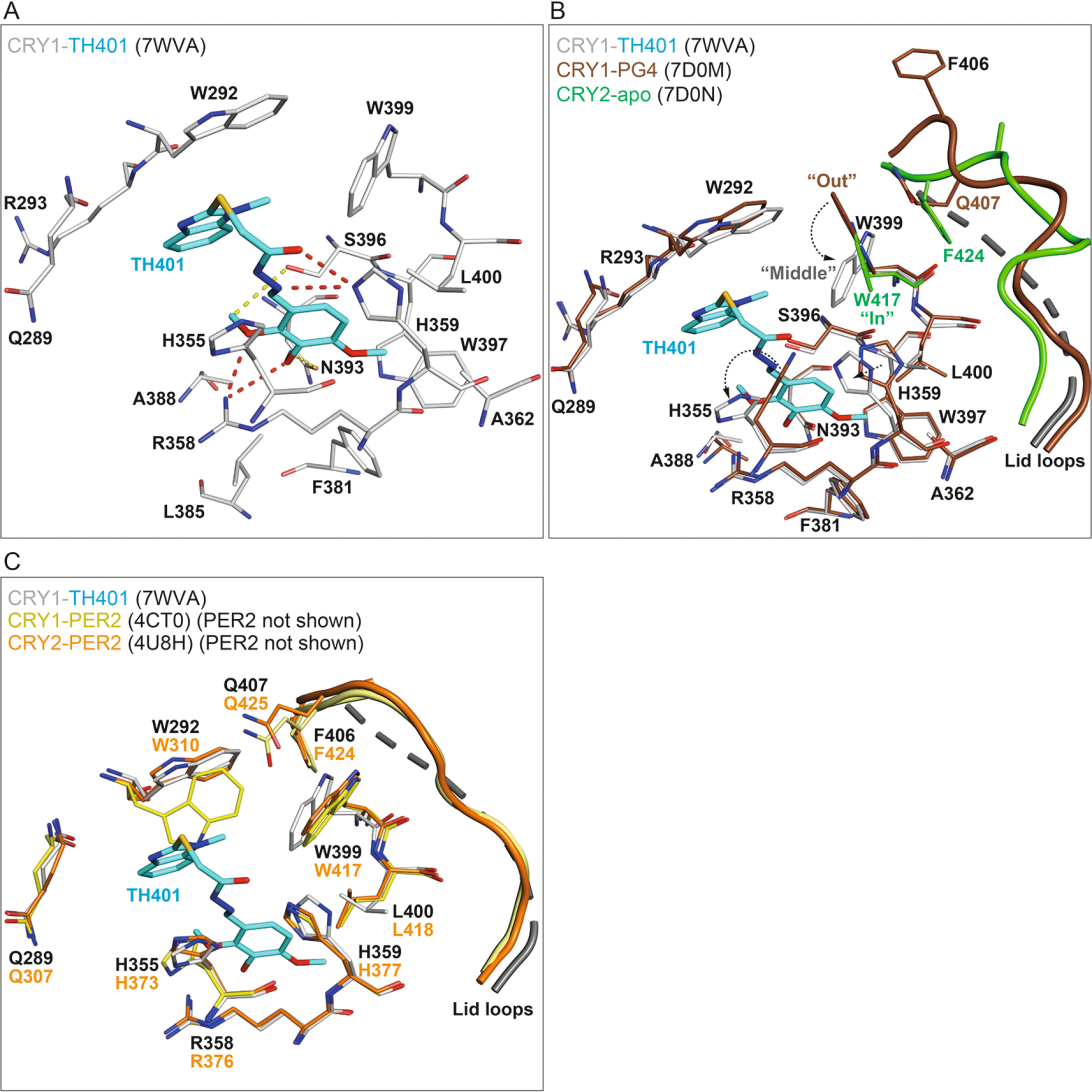
Crystal structure of CRY1-TH401 (PDB: 7WVA). (A) The binding mode of TH401 in CRY1. Flavin adenine dinucleotide (FAD) pocket residues (white) that interact with TH401 (cyan) are shown. Hydrogen bonds and C–H hydrogen bonds are represented by red and yellow dashes, respectively. (B) TH401 induced conformational changes in the FAD pocket of CRY1. Superposition of CRY1-TH401 (white-cyan) onto CRY1-PG4 (brown) (PDB: 7D0M; PG4, a non-biological tetraethylene glycol cryoprotectant, is not shown) and CRY2-apo (green) (PDB: 7D0N). Only the gatekeeper–lid loop interface is shown in CRY2-apo for simplicity. The binding of TH401 resulted in the repositioning of the gatekeeper W399 from an intrinsic “out” conformation in CRY1-PG4 (apo-like structure) to a “middle” conformation in CRY1-TH401. The W399 “middle” conformation resulted in the loss of an NH–aryl interaction between W399 and Q407 at the gatekeeper–lid loop interface in CRY1-PG4. Additional flexibility in the lid loop of CRY1 with bound TH401 meant the lid loop was not built into the crystal structure and its predicted structure is represented as dashed lines as modeled by Pymol. The intrinsic “in” conformation of the gatekeeper W417 in CRY2 would require a smaller conformational change to adopt a “middle” position than the intrinsic “out” conformation of W399 in CRY1. (C) Superposition of CRY1-TH401 (white-cyan) onto CRY1-PER2 (yellow) (PDB: 4CT0) and CRY2-PER2 (orange) (PDB: 4U8H). PER2 is not shown for simplicity. The binding mode of TH401 looks compatible with the key FAD pocket residues H355 and W399 in CRY1-PER2, corresponding to CRY2-PER2 residues H373 and W417, respectively.

To obtain insights into the regulatory effects of TH401 on CRY proteins, we determined the crystal structure of CRY1(PHR) in complex with TH401 at a resolution of 2.05 Å ( Table 1) (PDB ID: 7WVA). The overall protein fold was highly similar to previously published CRY1 structures. ^13^^–^^15^^,^^33^^–^^35^ With regard to the binding mode of TH401, the 1-methylbenzimidazole moiety formed hydrophobic interactions with W292, R293 and W399, as well as additional offset π-stacking with W292 ( Figure 3A). The trimethoxyphenyl moiety formed multiple hydrophobic interactions with residues R358, A362, F381, L385, A388, W397 and L400. Oxygen atoms in two methoxy groups ( *ortho* and *meta*) formed hydrogen bonds with the guanidinium group of R358, while methyl groups in two methoxy groups ( *ortho* and *meta*) formed C–H hydrogen bonds with N393 and S396 ( Figure 3A). One notable difference in the binding mode of TH401, compared to almost all other CRY-interacting compounds, was the absence of a canonical H-bond between the linker (connecting the methylbenzimidazole and trimethoxyphenyl moieties) and S396. Instead, H359 interacted with the sulfanylacetohydrazide linker by forming two hydrogen bonds, one with the hydrazide carbonyl and the other with a hydrazide nitrogen ( Figure 3A).

**Table 1.** Data collection and refinement statistics. Values in parentheses are for the highest resolution shell. Root mean square (R,m.s.); correlation coefficient (CC).

	CRY1-TH401 (7WVA)
**Data collection**	
Space group	P2 _1_2 _1_2 _1_
Cell dimensions	
a, b, c (Å)	44.9, 78.2, 132.8
α, β, γ (°)	90, 90, 90
Resolution (Å)	2.05 (2.16-2.05)
R _merge_	0.077 (0.475)
I/σ(I)	13.7 (3.0)
CC _1/2_	0.999 (0.891)
Completeness (%)	99.9 (99.7)
Redundancy	7.4 (7.2)
**Refinement**	
Resolution (Å)	66.41-2.051
No. reflections [unique]	221735 [30079]
R _work_/R _free_	0.1897/0.2204
No. atoms	3840
Protein	3640
Ligand/ion	29
Water	171
R.m.s. deviations	
Bond lengths (Å)	0.007
Bond angles (°)	0.779
Ramachandran	
Favored (%)	97.67
Allowed (%)	2.33
Outliers (%)	0
Average B-factors	
Protein	37.08
Ligand	33.38
Solvent	37.42

TH401 binding was compatible with the intrinsic conformations of most FAD (flavin adenine dinucleotide) pocket residues of CRY1; however, a notable difference was observed in the conformation of the gatekeeper W399, and steric restraint was imposed on the possible rotamer positions of H355 ( Figure 3B). W399 underwent a sizeable conformational change from an intrinsic “out” position to a “middle” conformation to form a hydrophobic interaction with the methyl group of the 1-methylbenzimidazole moiety, and H355 adopted a forward-facing rotamer, similar to an alternate conformer that was observed in the CRY1-apo structure ^13^ (PDB ID: 6KX4). The lid loop was disordered in CRY1-TH401, most likely because the intrinsic W399–Q407 gatekeeper–lid loop interface was disrupted by TH401-induced repositioning of W399 ( Figure 3B). Overall, the binding mode of TH401 appears less compatible with the FAD pocket in CRY1 than CRY2, which supports the slightly lower interaction of CRY1 over CRY2 in thermal shift assays ( Figure 2E and F).

Our structural data showed that TH401 binding to CRY1 induced a sizeable conformational change in the gatekeeper W399. Isoform-specific gatekeeper conformations that mediate distinct gatekeeper–lid loop interfaces in CRY1 and CRY2 have been implicated in the potential regulation of compound isoform-selectivity. ^35^^,^^36^ Interestingly, the TH401-induced “middle” gatekeeper conformation in the CRY1-TH401 structure would appear to be more energetically favorable in CRY2, because only a small conformational change (W417 from “in” to “middle”; CRY2 W417 corresponds to CRY1 W399) would be required ( Figure 3B). In contrast, CRY1 W399 would need to rotate much further from an “out” to a “middle” conformation. Furthermore, the NH–aryl interaction between W399 and Q407 in CRY1 has more favorable free binding energy than the stacking interaction of W417 and F424 in CRY2, ^35^ which may result in CRY1 W399 being less flexible than CRY2 W417. These structural observations correlate to the slightly lower preferential interaction of TH401 with CRY1 compared to CRY2 in thermal shift assays. In contrast, however, TH401 displayed a very low level of isoform preference in functional assays ( Figure 2C and D). This disparity may be due to higher repressor activity of CRY1 over CRY2, ^37^^–^^40^ resulting in the similar functional effects of TH401 on both isoforms, despite its preferential interaction with CRY2. In addition to the gatekeeper and lid loop, a flexible region downstream of the PHR known as the CRY C-terminal tail (CCT) has been associated with compound selectivity. ^13^ In *Drosophila* CRY, the residue H378, corresponding to mouse CRY1 H355, has been shown to regulate CCT interaction with the FAD pocket via a conformational change. ^41^^–^^43^ Both W399 and H355 in CRY1-TH401 underwent large conformational changes, compared to CRY1-apo structures ^35^ ( Figure 3B), and the lid loop was disordered as a result of W399-repositioning. These changes could affect CCT interaction for functional changes.

CRYs form large complexes in both the cytoplasm and nucleus, and PER2, a primary CRY-interacting protein, changes the conformations of key FAD pocket residues, including the gatekeeper W399, as well as the lid loop ^44^ (PDB ID: 4CT0). Interestingly, the conformations of the gatekeeper W399 and H355 in the CRY1-TH401 crystal structure are very similar to those in CRY1/2-PER2 complex structures ^44^^,^^45^ ( Figure 3C) (PDB IDs: 4CT0; and 4U8H). The conformation of W292 in CRY1-PER2 would form a steric clash with the methylbenzimidazole of TH401; however, W292 is very flexible and can accommodate compounds by easily adopting a different rotamer. ^35^ Therefore, TH401 may be able to bind to CRY1 and CRY2 equally when they are complexed with PER2, resulting in similar potency against both isoforms.

**Figure 4.**
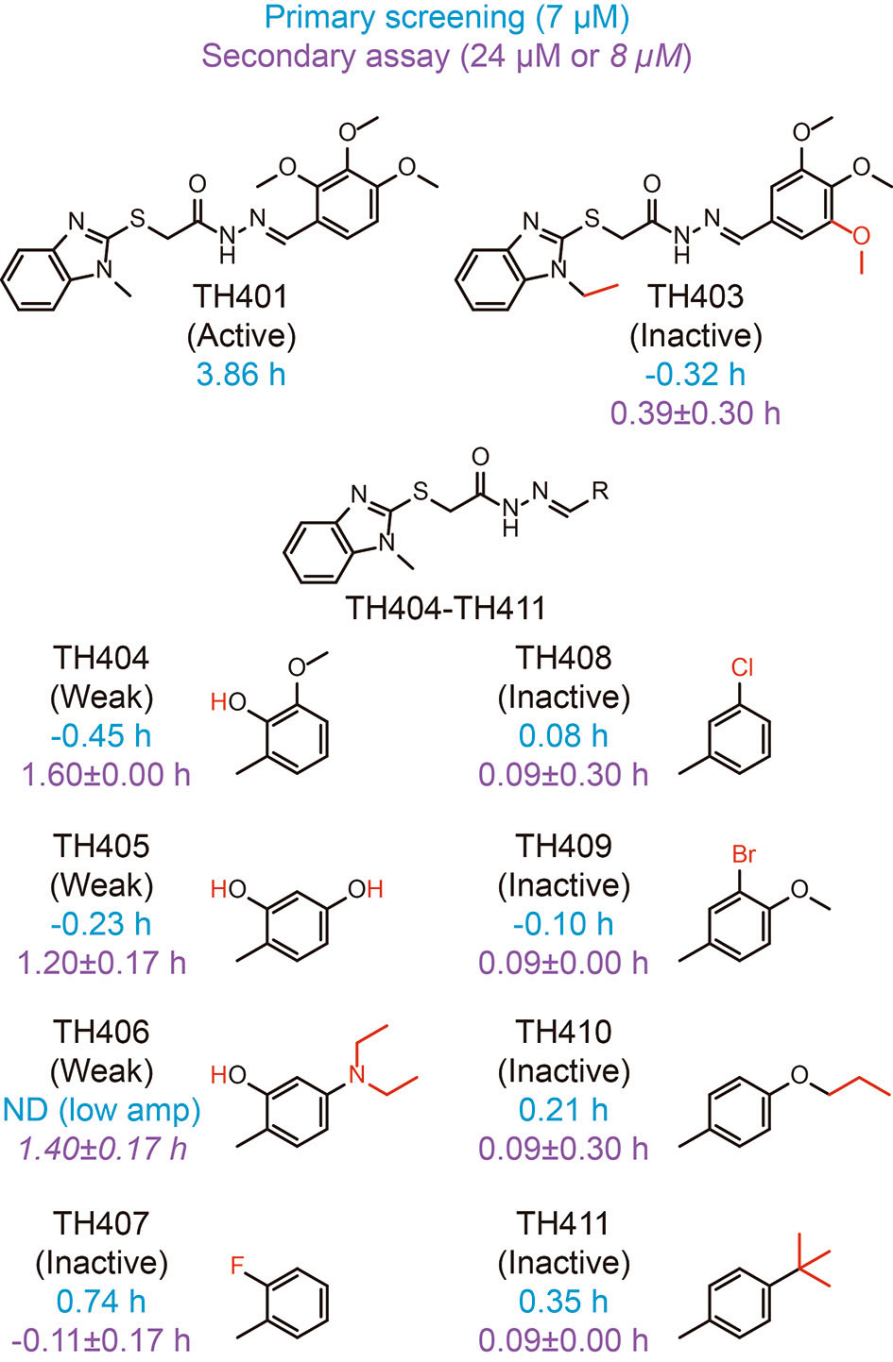
Period-lengthening activities of TH401 derivatives. Changes in the circadian period of *Bmal1-dLuc* U2OS cells compared to a dimethyl sulfoxide (DMSO) control in primary screening (tested at 7 μM; *n* = 1) and a secondary assay (tested at 24 μM; mean±SD, *n* = 3 biologically independent samples) are shown in blue and purple, respectively, with chemical structures. TH406 caused low amplitude with unreliable period estimation (ND, not determined) in primary screening and the secondary assay at 24 μM, and were therefore tested at 8 μM (shown in italics). Modified part of the compound is shown in red.

To further characterize the CRY2-TH401 interaction, we searched for TH401 derivatives in the compound library used for primary screening of circadian clock modulators and checked their activity in the screen ( Figure 4, blue). ^30^ Because the derivatives TH403-TH411 showed almost no effect on circadian period in the screen at 7 μM (using 1 mM working stock compounds), we obtained these compounds from the original 10 mM stock of the library and analyzed their activity in a circadian assay using human *Bmal1-dLuc* U2OS cells at 24 or 8 μM ( Figure 4, purple). ^30^ Extension of the methyl group of 1-methylbenzimidazole together with replacement of the *ortho*-methoxy group of trimethoxyphenyl to *meta* (TH403) caused a loss of activity, consistent with the interactions of the methyl group with W399, and the *ortho*-methoxy group with R358 and S396 ( Figure 3A). Modifications to the trimethoxy groups of the trimethoxyphenyl resulted in either weak activity (TH404-TH406) or inactivity (TH407-TH411), supporting their interactions with R358, S396, and N393, as well as A362, F381, L385, A388, W397, and L400. The weak activities of TH404-TH406 suggested that an interaction of the *ortho*-hydroxy group with R358 can support activity. Therefore, CRY2-TH401 interactions in the crystal structure are consistent with activity in cells.

## Conclusion

We have discovered that TH401 provides a new chemical scaffold, methylbenzimidazole, for CRY regulation by targeting both CRY1 and CRY2. Cell-based phenotypic screens of circadian clock modulators resulted in the identification of small-molecule activators of CRY proteins. In addition to this approach, CRY inhibitors have been identified through a cell-based screen of E-box-mediated transcription. 2-ethoxypropanoic acid derivatives target both CRY isoforms and inhibit their repressive function, enhancing E-box-mediated transcription. ^46^^,^^47^ Furthermore, a recent study showed that structure-based drug design could be another useful approach to find CRY1 modulators. ^48^ In order to obtain further insights into the mechanisms of action of these small-molecules, it is necessary to determine the crystal structures in complex with CRY proteins. Identification of new CRY modulators and their characterization will facilitate the understanding and regulation of CRY protein functions in gene expression, ^49^ metabolism, ^7^^,^^10^^,^^11^^,^^13^^,^^50^^,^^51^ cancer, ^12^^,^^52^^–^^54^ and sleep-wake rhythms, ^55^^–^^57^ ultimately leading to the discovery of therapeutic agents for circadian clock-related diseases.

## Data availability

### Underlying data

The X-ray crystal structure of CRY1-TH401 was deposited into the Protein Data Bank with the accession number 7WVA.

Figshare: Yagi et al. Figure data. https://doi.org/10.6084/m9.figshare.20431692. ^30^

This project contains the following underlying data:

• Figure 1B. csv (Luminescence rhythms of *Bmal1-dLuc* and *Per2-dLuc* U2OS cells in the presence of various concentrations of TH401 ( *n* = 2))
• Figure 1C. csv (Changes in period ( *n* = 6))
• Figure 1D. csv (Changes in luminescence intensity ( *n* = 6))
• Figure 1E. csv (Changes in cellular ATP levels after treatment with various concentrations of TH401 ( *n* = 4))
• Figure 2A. csv (Inhibitory effect of Longdaysin and TH401 on CKIδ activity *in vitro* ( *n* = 1))
• Figure 2B. csv (Changes in the half-lives of CRY-luciferase fusion proteins (CRY1-LUC and CRY2-LUC) relative to LUC in the presence of various concentrations of TH401 ( *n* = 2))
• Figure 2C. csv (Changes in *Per2::Luc* knock-in reporter activity in wild type, *Cry1/Cry2* double knockout, *Cry1* knockout, and *Cry2* knockout fibroblasts ( *n* = 4–8))
• Figure 2D. csv (Changes in the cellular circadian period of *Bmal1-Eluc* reporter rhythms in *Cry1/Cry2* double knockout fibroblasts rescued with CRY1 and CRY2 ( *n* = 3–6))
• Figure 2E. csv (Changes in the protection of CRY1 and CRY2 proteins from thermal denaturation in HEK293T cells ( *n* = 4))
• Figure 2F. csv (Changes in denaturing temperatures of recombinant CRY1(PHR) and CRY2(PHR) *in vitro* ( *n* = 2))
• Figure 4. csv (Changes in the circadian period in primary screening (tested at 7 μM; *n* = 1) and a secondary assay (tested at 24 or 8 μM; *n* = 3))

Data are available under the terms of the Creative Commons Zero “No rights reserved” data waiver (CC0 1.0 Public domain dedication).

## Accession numbers

Protein Data Bank:

• Crystal structure of mouse Cryptochrome 1 in complex with TH401 compound. Accession number: 7WVA. https://doi.org/10.2210/pdb7WVA/pdb.
• Crystal structure of mouse CRY1 with bound cryoprotectant. Accession number: 7D0M. https://doi.org/10.2210/pdb7D0M/pdb.
• Crystal structure of mouse CRY2 apo form. Accession number: 7D0N. https://doi.org/10.2210/pdb7D0N/pdb.
• Crystal Structure of Mouse Cryptochrome1 in Complex with Period2. Accession number: 4CT0. https://doi.org/10.2210/pdb4CT0/pdb.
• Crystal Structure of Mammalian Period-Cryptochrome Complex. Accession number: 4U8H. https://doi.org/10.2210/pdb4U8H/pdb.
• Crystal structure of mouse Cryptochrome 1 apo form. Accession number: 6KX4. https://doi.org/10.2210/pdb6KX4/pdb.
