# Supplementary material for: A methylbenzimidazole derivative regulates mammalian circadian rhythms by targeting Cryptochrome proteins
Source: F1000Res. 2022 Oct 31;11:1016. Originally published 2022 Sep 7. [Version 2] doi: 10.12688/f1000research.124658.2 (PMC9523283; doi:10.12688/f1000research.124658.2)
Supplement: Peer review history

# Peer review: Reviewer response for version 2

**DOI:** 10.5256/f1000research.139304.r154542

**Published:** 2022-11-03

**Authors:** Eric Zhang

**Affiliations:** 1 National Institute of Biological Sciences (NIBS), Beijing, China

This revised version has addressed all my concerns.

Is the work clearly and accurately presented and does it cite the current literature?

Yes

If applicable, is the statistical analysis and its interpretation appropriate?

Yes

Are all the source data underlying the results available to ensure full reproducibility?

Yes

Is the study design appropriate and is the work technically sound?

Yes

Are the conclusions drawn adequately supported by the results?

Yes

Are sufficient details of methods and analysis provided to allow replication by others?

Yes

Reviewer Expertise:

Circadian clock

I confirm that I have read this submission and believe that I have an appropriate level of expertise to confirm that it is of an acceptable scientific standard.

# Peer review: Reviewer response for version 1

**DOI:** 10.5256/f1000research.136879.r149801

**Published:** 2022-09-29

**Authors:** Katja Lamia

**Affiliations:** 1 Department of Molecular Medicine, Scripps Research Institute, La Jolla, CA, USA

Moeri Yagi and colleagues report the identification of a chemical stabilizer of CRY1 and CRY2 based on a methylbenzimidazole scaffold, which is different from the scaffolds of previously reported CRY-stabilizing compounds. They present well designed experiments that persuasively show that this compound, TH401, interacts directly with both CRY1 and CRY2 at their FAD-binding pockets and thereby leads to their stabilization and lengthening of circadian period in cells.

 I have one minor suggestions to improve clarity: In the introduction, the sentence “…compounds that control clock function provide a powerful and useful tool in the drug discovery of such diseases” is unclear. Do the authors mean “… compounds that control clock function provide a powerful and useful tool in drug discovery related to diseases that are impacted by circadian disruption”?

Is the work clearly and accurately presented and does it cite the current literature?

Yes

If applicable, is the statistical analysis and its interpretation appropriate?

Yes

Are all the source data underlying the results available to ensure full reproducibility?

Yes

Is the study design appropriate and is the work technically sound?

Yes

Are the conclusions drawn adequately supported by the results?

Yes

Are sufficient details of methods and analysis provided to allow replication by others?

Yes

Reviewer Expertise:

circadian biology, cryptochrome proteins

I confirm that I have read this submission and believe that I have an appropriate level of expertise to confirm that it is of an acceptable scientific standard.

# Response

**Published:** 2022-09-30

**Authors:** Tsuyoshi Hirota

**Affiliations:** Nagoya University, Japan

**Reviewer #2:**

*Moeri Yagi and colleagues report the identification of a chemical stabilizer of CRY1 and CRY2 based on a methylbenzimidazole scaffold, which is different from the scaffolds of previously reported CRY-stabilizing compounds. They present well designed experiments that persuasively show that this compound, TH401, interacts directly with both CRY1 and CRY2 at their FAD-binding pockets and thereby leads to their stabilization and lengthening of circadian period in cells.*

*I have one minor suggestions to improve clarity: In the introduction, the sentence “…compounds that control clock function provide a powerful and useful tool in the drug discovery of such diseases” is unclear. Do the authors mean “… compounds that control clock function provide a powerful and useful tool in drug discovery related to diseases that are impacted by circadian disruption”?*

 We thank the reviewer for the insightful comment. We are very pleased to hear that the reviewer approved the publication of this manuscript. We agree with the reviewer and modified the sentence accordingly.

# Peer review: Reviewer response for version 1

**DOI:** 10.5256/f1000research.136879.r149802

**Published:** 2022-09-21

**Authors:** Eric Zhang

**Affiliations:** 1 National Institute of Biological Sciences (NIBS), Beijing, China

In this article, Moeri Yagi and co-authors describe the characterization of a small molecule TH401 which stabilizes both isoforms of the clock protein Cryptochrome (CRY1 and CRY2) and lengthens the period of circadian rhythms in cellular models. The co-crystal structure of CRY1 in complex with TH401 was obtained and compared with the structure of CRY1 and CRY2, explaining the slight preference of TH401 to CRY2. Several TH401 derivatives were further tested for their period-lengthening activities, confirming the proposed mechanism of CRY-TH401 interaction. As a methylbenzimidazole derivative, TH401 provides a new scaffold for CRY modulators, which will not only enable a better understanding of the structure of CRY, but also contribute to the discovery of therapeutics against circadian clock-related diseases. In general, this manuscript is well-written, and I recommend its publication after a minor revision.

**Specific comments:**

1. In the first paragraph on page 9, the conclusion “the binding mode of TH401 appears less compatible with the FAD pocket in CRY1 than CRY2” is drawn before the structure of FAD pocket in CRY2 is elucidated, which is somehow confusing.
2. In the third paragraph on page 10, the authors state “To further characterize the CRY2-TH401 interaction…” at the beginning and “CRY2-TH401 interactions in the crystal structure are consistent with activity in cells” in the end. However, the experiments using *Bmal1-dLuc* U2OS cells can not reflect the interaction of TH401 derivatives with CRY1 and CRY2 separately. It might be better to change “CRY2-TH401” into “CRY-TH401”.
3. Although several TH401 derivatives have been tested for their period-lengthening activities, no mutants of CRY1 or CRY2 were tested for their interactions with TH401. Rescue assay in *Cry1/Cry2*double knockout fibroblasts with CRY mutants carrying point mutations in TH401 binding residues may further confirm the proposed mechanism of CRY-TH401 interaction and is therefore recommended.
4. The hyphen should not be added between “small molecule” when it is used as a noun. This has appeared three times: “Although small-molecules that…” in Abstract, “…identified a new small-molecule TH401 that…” in Abstract and “…action of these small-molecules” in Conclusion.
5. Table 1 legend: “R,m.s.” should be “R.m.s.”

Is the work clearly and accurately presented and does it cite the current literature?

Yes

If applicable, is the statistical analysis and its interpretation appropriate?

Yes

Are all the source data underlying the results available to ensure full reproducibility?

Yes

Is the study design appropriate and is the work technically sound?

Yes

Are the conclusions drawn adequately supported by the results?

Yes

Are sufficient details of methods and analysis provided to allow replication by others?

Yes

Reviewer Expertise:

Circadian clock

I confirm that I have read this submission and believe that I have an appropriate level of expertise to confirm that it is of an acceptable scientific standard.

# Response

**Published:** 2022-09-30

**Authors:** Tsuyoshi Hirota

**Affiliations:** Nagoya University, Japan

**Reviewer #1:**

*In this article, Moeri Yagi and co-authors describe the characterization of a small molecule TH401 which stabilizes both isoforms of the clock protein Cryptochrome (CRY1 and CRY2) and lengthens the period of circadian rhythms in cellular models. The co-crystal structure of CRY1 in complex with TH401 was obtained and compared with the structure of CRY1 and CRY2, explaining the slight preference of TH401 to CRY2. Several TH401 derivatives were further tested for their period-lengthening activities, confirming the proposed mechanism of CRY-TH401 interaction. As a methylbenzimidazole derivative, TH401 provides a new scaffold for CRY modulators, which will not only enable a better understanding of the structure of CRY, but also contribute to the discovery of therapeutics against circadian clock-related diseases. In general, this manuscript is well-written, and I recommend its publication after a minor revision.*

 We thank the reviewer for the insightful comments. We are very pleased to hear that the reviewer recommends the publication of this manuscript. Our point-by-point responses are listed below. We believe that the changes have clarified our manuscript.

**Comment 1:** *In the first paragraph on page 9, the conclusion “the binding mode of TH401 appears less compatible with the FAD pocket in CRY1 than CRY2” is drawn before the structure of FAD pocket in CRY2 is elucidated, which is somehow confusing.*

 We agree with the reviewer and modified the sentence to "Overall, the binding mode of TH401 is not fully compatible with the intrinsic FAD pocket in CRY1-apo and induces conformational rearrangement of key pocket residues for a favorable interaction."

**Comment 2:** *In the third paragraph on page 10, the authors state “To further characterize the CRY2-TH401 interaction…” at the beginning and “CRY2-TH401 interactions in the crystal structure are consistent with activity in cells” in the end. However, the experiments using Bmal1-dLuc U2OS cells can not reflect the interaction of TH401 derivatives with CRY1 and CRY2 separately. It might be better to change “CRY2-TH401” into “CRY-TH401”.*

 Thank you for raising this point. We changed “CRY2-TH401” to “CRY-TH401”.

**Comment 3:** *Although several TH401 derivatives have been tested for their period-lengthening activities, no mutants of CRY1 or CRY2 were tested for their interactions with TH401. Rescue assay in Cry1/Cry2double knockout fibroblasts with CRY mutants carrying point mutations in TH401 binding residues may further confirm the proposed mechanism of CRY-TH401 interaction and is therefore recommended.*

 This is an interesting point that we would like to address in our future studies. Thank you for your suggestion.

**Comment 4:** *The hyphen should not be added between “small molecule” when it is used as a noun. This has appeared three times: “Although small-molecules that…” in Abstract, “…identified a new small-molecule TH401 that…” in Abstract and “…action of these small-molecules” in Conclusion.*

 Thank you for pointing this out. We removed the hyphen.

**Comment 5:** *Table 1 legend: “R,m.s.” should be “R.m.s.”*

 Thank you for pointing this out. It was fixed.
